# Understanding the Emergence of Comorbidity between Problematic Online Gaming and Gambling: A Network Analysis Approach

**DOI:** 10.3390/brainsci14090929

**Published:** 2024-09-18

**Authors:** Marta Błoch, Błażej Misiak

**Affiliations:** Department of Psychiatry, Wroclaw Medical University, 50-367 Wroclaw, Poland; blazej.misiak@umw.edu.pl

**Keywords:** internet addiction, behavioral addiction, network analysis, directed acyclic graph

## Abstract

Background/Objectives: Problematic online gaming and gambling tend to co-occur. The exact mechanisms underlying this phenomenon and the potential effects of gender differences remain unknown. This study aimed to identify the early clustering patterns of problematic online gaming and gambling in a community sample of young adults without a lifetime history of psychiatric treatment. Methods: Data were collected through an online survey and analyzed using partial correlations and Bayesian networks. Results: Altogether, 1441 individuals (aged 18–40 years, 51.4% females) participated in the survey. Both problematic online behaviors were weakly interrelated, suggesting that they serve as distinct constructs. Men’s networks appeared to be more complex and had significantly higher global connectivity. Moreover, men and women differed with respect to the specific nodes that bridged both constructs. In men, the bridge nodes were “being criticized because of betting or being told about gambling problems”, “loss of previous interests due to gaming”, “deceiving other people because of gaming”, and “health consequences of gambling”. Among women, the bridge nodes were “feeling guilty because of gambling”, “loss of previous interests because of gaming”, “social consequences of gaming”, and “continued gaming problems with other people”. In men, the strongest edge was found between “borrowing money/selling anything to gamble” and “financial problems because of gambling”, while in women, the strongest edge appeared between “betting more than afforded to be lost” and “tolerance symptoms of gambling”. Conclusions: The findings indicate that problematic online gaming and gambling tend to emerge in different ways among men and women. Therapeutic interventions should be planned considering gender differences.

## 1. Introduction

Researchers and clinicians still disagree on the concept of behavioral disorders. Some consider them to be specific brain disorders with neurobiological alterations similar to those observed in substance addiction [1,2,3]. In contrast, others express concerns about the excessive pathologizing of common, everyday behaviors [4]. This is particularly evident in the case of a range of online behaviors, which have appeared to be the topic of an ongoing debate in public health in recent years [5]. Indeed, in the era of constant Internet access, it is difficult to find a cut-off point for its ‘excessive’ or ‘addictive’ use. In this regard, the term problematic online behaviors (POBs) has emerged and concentrates on the harm that these disorders cause to an individual’s social, occupational, or psychological functioning [6,7]. Two out of several POBs described, i.e., problematic online gambling and problematic online gaming, are well examined and have documented health consequences [8,9,10,11,12]. Undoubtedly, they mainly affect adolescents and young adults, so the phenomenon is set to grow and pose a major public health challenge. In this population, the presence of academic problems increases the likelihood that they will not only use the Internet to gamble but also become problem gamblers [13,14,15]. This is particularly important given its applicability to the field of psychoeducation.

The concept of distinguishing addiction from problematic behavior has also been addressed in the latest International Statistical Classification of Diseases and Related Health Problems (11th ed.; ICD-11), where gambling and gaming disorders are included in the chapter entitled ‘Disorders due to addictive behaviors’. In addition, separate diagnoses, hazardous gambling and hazardous gaming, have been placed among ‘Problems associated with health behaviors’. The ICD-11 has also highlighted the growing problem of POBs by differentiating the ‘online’ subtypes of these two disorders [16].

Different forms of POBs tend to co-occur [17,18]. Several studies have focused on the comorbidity of gambling and gaming and have shown an association between the two: those who report gambling are more likely to report gaming and vice versa [19,20,21]. The causes of this comorbidity phenomenon are still unclear. Empirical evidence suggests that common elements of different symptoms may facilitate a cycle of reciprocity, increasing the risk of switching from one addictive behavior to another. In addition, individuals may seek satisfaction through alternative addictive behaviors while trying to discontinue their primary addiction [22,23]. These considerations may be important for problematic online gaming and gambling. Previous studies have also identified shared risk factors for gambling and gaming disorders. Individuals with high levels of novelty and sensation seeking, impulsivity, and dependence on rewards and punishments are more likely to experience both conditions [24,25,26,27]. Delfabbro and King further suggest that the ‘digital convergence’ of these activities facilitates the coexistence of symptoms of both problems [28]. Moreover, many video games contain structural elements that share similarities with gambling systems, e.g., loot boxes [19,29], which may present similar risk factors for problematic gambling among vulnerable individuals. However, in recent research, there is less evidence to support a direct relationship between problematic gaming and gambling [30]. 

In seeking to understand the complex mechanisms underlying this comorbidity, it is worth considering the importance of gender differences [31]. Many characteristics are well-established for substance use disorders but are less understood for behavioral disorders [17]. Historically, gambling and gaming have been considered predominantly men’s activities, but it is increasingly becoming recognized that women are also likely to engage in gambling and gaming [32]. Differences in emotional sensitivity to changes in the stress system between women and men might be of importance [33,34,35]. The higher rates among men are explained by their higher risk-taking, stimulation-seeking, impulsive, competitive, and aggressive traits compared to women [36,37,38,39,40]. In other words, men gamble or play games more often to enhance their positive mood or as thrilling activities. Women tend to use this as a coping mechanism for emotional distress and negative emotions [34]. Men also choose more strategic forms of gambling and gaming, which can be more addictive [37]. It appears that compared with substance use disorders, more complex mechanisms involving biological and social factors are likely to account for gender differences in behavioral disorders, which may also reflect differences in the weighting of criteria for these disorders between women and men [17,39,41,42]. Notably, most of the research to date has looked at their ‘offline’ forms and has been conducted in predominantly male population samples [36,43,44]. However, studies performed in the general population samples might provide valuable insights into how men and women differ in their behaviors and motivations regarding these activities.

Recent research on POBs confirms that male gender is indeed predictive of problematic online gaming [11,45,46]. It has been shown that women are more likely to engage in online gambling activities compared to men, who might be more likely to engage in offline forms of gambling [31,45,47]. The search for the cause of this phenomenon is still ongoing. One theory is that women, for whom gambling used to be less socially acceptable, are now more vulnerable because of the increasing accessibility of online gambling and its complete anonymity [38]. The engagement of women in Internet gambling may explain why recent studies from many jurisdictions show a rapid increase in problematic gambling among women, particularly those at a younger age [48]. However, these causes are still being investigated.

Although it has been observed that problematic online behaviors tend to co-occur, specific mechanisms of their interactions remain unknown [18]. The current conceptualization of psychopathology suggests that mental disorders are a group of symptoms and can, therefore, be explained by a latent (or unobserved) construct [49]. Using network analysis, one can estimate the relationship between specific symptoms using a graphical model [50]. Rather than merely assessing the comorbidity of different constructs, network analysis enables the study of symptoms within and between disorders [51]. Such data can be particularly useful because symptoms that co-occur in different disorders, also known as bridge nodes, can provide insights into the mechanisms underlying the development of comorbidities [50]. Network analysis also shows the centrality indices, i.e., symptoms/behaviors that are the most important in the network structure [52]. 

Using network analysis, Zarate et al. found several connections between POBs and addictive behaviors related to substance use, with gambling showing the highest centrality [18]. However, this study did not consider the effects of gender differences and did not specifically focus on problematic online gaming or gambling. To date, only two studies have investigated problematic gambling with respect to gender. The first assessed the social and financial aspects of gambling rather than addiction as a pathological construct [53]. The second one used a sample of help-seekers, which makes it difficult to draw conclusions about the general population [54]. Another study on gambling disorder and sexual addiction provided valuable information on the distinctiveness of both constructs and on the differences in the complexity of symptom networks according to gender [55]. Taking into consideration existing gaps in the field, the aims of the present study were twofold. First, it aimed to provide insights into the dynamic interactions between the symptoms of problematic online gambling and gaming using partial correlation (undirected) and Bayesian (directed) networks in a community sample of young adults without a history of psychiatric treatment. Second, the study aimed to recognize potential gender differences in the early comorbidity patterns of problematic online gaming and gambling.

## 2. Materials and Methods

### 2.1. Participants

Participants were selected through an online platform commonly used for research surveys. The inclusion criteria were age between 18 and 40 years and no prior history of psychiatric treatment. History of psychiatric treatment was determined using one item: “Have you ever received any psychiatric treatment?”. The recruitment methods were designed to ensure that the demographic characteristics of the participants closely resembled those of the general Polish population, particularly in terms of age and gender. The study protocol was approved by the Bioethics Committee of Wroclaw Medical University, Wroclaw, Poland (approval number: 240/2024). Before proceeding with the survey, participants were informed about the confidentiality and the anonymous nature of data collection. All participants provided informed consent. 

### 2.2. Measures

Participants were asked to complete a demographic questionnaire and a self-report questionnaire. Demographic information included age, gender, education level, employment status, place of residence, relationship status, and monthly income. Next, specific questionnaires recording the symptoms of problematic online gaming and gambling were administered.

#### 2.2.1. Problematic Online Gaming 

To measure problematic online gaming, we used the validated Polish version of the Internet Gaming Disorder Scale-Short-Form (IGDS9-SF), based on the DSM-5 criteria [56]. Specific items are scored on a five-point scale (i.e., between 1—‘never’ and 5—‘very often’). The total score ranges from 9 to 45. Higher scores indicate a higher level of problematic online gaming. A cut-off for the total score of at least 32 for problematic online gaming was used [57]. The Cronbach’s alpha of the IGDS9-SF study was 0.92 in the present study.

#### 2.2.2. Problematic Online Gambling

Problematic online gambling was assessed using the Polish version of the Problem Gambling Severity Index (PGSI) [58], which is based on the Canadian Problem Gambling Index [59]. Although this questionnaire is based on criteria from the DSM-IV, it is still an accepted and widely used tool [58]. It is the only test for this disorder that has been validated and adapted for use in the Polish language. This tool consists of nine items scored on a 4-point scale (specific responses range between 0—‘never’ and 3—‘almost always’). A total score of ≥8 has been proposed as the threshold score for positive screening for problematic online gambling [58]. Cronbach’s alpha for the PGSI was 0.96 in the present study.

### 2.3. Procedure

The study was conducted in March 2024 in a community sample from Poland using computer-assisted web interviews. Eligible individuals were provided with a survey link containing self-report questionnaires. Responses that were incomplete or significantly deviated from the mean were filtered out by the platform.

### 2.4. Statistical Analysis

First, a non-directed partial correlation network using Gaussian graphical models was analyzed. The least absolute shrinkage and selection operator (LASSO) based on the Extended Bayesian Information Criterion (EBIC) was implemented [50]. This approach allows us to reduce the number of very weak correlations by converting their coefficients to zero values. The output of this analysis is a network of variables, shown as nodes that are connected with edges. Thicker and more saturated edges correspond to stronger partial correlation coefficients. To assess the importance of specific nodes in connecting communities (i.e., problematic online gaming and gambling), the bridge expected influence was estimated. Higher values of this metric indicate a greater ability of a specific node to activate symptoms across communities. As opposed to other bridge centrality metrics, the bridge expected influence takes into consideration the presence of both positive and negative edges [60]. As proposed previously, the top 10% of the nodes with the greatest bridge expected influence were interpreted as bridge nodes [61]. The stability of the edge weights and the bridge expected influence were assessed using a case-drop bootstrapping procedure. This approach allows us to calculate the correlation stability coefficient (CS-C), which should be higher than 0.25. In this part of the data analysis, we used the following R packages: *networktools* [61], *bootnet* [50], *qgraph* [62], and *mgm* [63]. 

Next, a comparison of women’s and men’s networks using the permutation test developed by van Borkulo et al. [64] was performed. Specifically, we used the network invariance test and the global expected influence invariance test. To adjust for multiple comparisons, the Benjamini-Hochberg correction was used. The results of these tests were considered significant if the *p*-value was lower than 0.05. This part of data analysis was carried out using the *networkcomparisontest* [64] implemented in the R studio. 

The final part of the data analysis was related to estimating the Bayesian network and visualizing directed acyclic graphs (DAGs) in men and women. Importantly, DAGs provide information about the directional probabilities between nodes [65]. The hill climbing algorithm based on the Bayesian Information Criterion (BIC) was applied to assess the goodness of fit. More frequently appearing edges show greater strength and directional probability. A non-parametric bootstrapping based on 5000 iterations was used to ensure the stability of the network. The resulting network was averaged to show the associations observed in more than 50% of the models. This part of the data analysis was performed using *bnlearn* [66] and *bnviewer* R packages [67]. We used the R code previously developed by Hunt et al. [65].

## 3. Results

### 3.1. General Characteristics of the Sample

Altogether, 2775 individuals were invited to participate in the survey. Among them, 659 individuals (23.8%) were excluded due to a lifetime history of psychiatric treatment. Moreover, 635 individuals (22.9%) declined to participate, and 40 individuals did not complete the whole survey (1.4%). Finally, 1441 individuals (51.9%) completed the survey (aged 29.5 ± 6.3 years, 51.4% females, Table 1). Most frequently, participants reported a higher education level (43.3%), full-time employment (52.9%), urban place of residence (63.7%), single marital status (41.1%), and monthly income of 750–1250 USD (39.2%). Positive screening for problematic online gaming and gambling was found in 2.1% and 6.0% of the participants, respectively.

### 3.2. Partial Correlations Network

Case-drop bootstrapping revealed sufficient correlations between bootstrap samples and the original data, indicating that the networks were stable. The CS-C value for the bridge expected influence was 0.36 (the same value in the case of men’s and women’s networks). In turn, the CS-C value for edges was 0.75 in men and 0.67 in women. 

**Figure 1:**
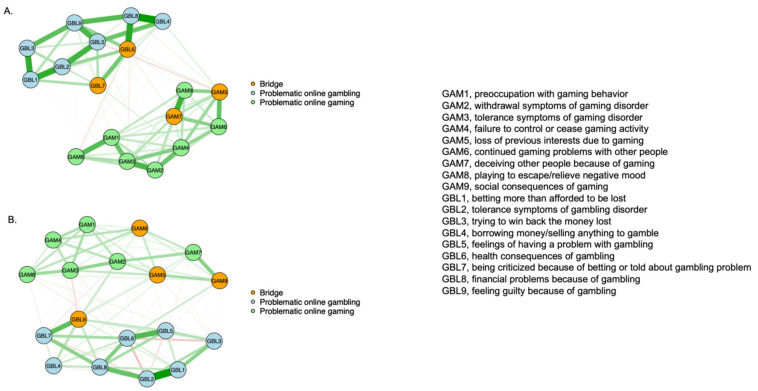
The partial correlation networks in men (**A**) and women (**B**). The network shows specific variables as nodes that are connected to the edges. Green nodes illustrate the characteristics of problematic online gaming, while blue nodes refer to problematic online gambling. Orange nodes are those with the top 10% bridge centrality metrics. Thicker and more saturated edges indicate stronger connections. Green edges correspond to positive associations, while red edges illustrate negative associations.

The networks analyzed in the present study are visualized in Figure 1A,B. Out of 153 possible edges, the men’s network had 107 non-zero edges (69.9%), while the women’s network showed 109 non-zero edges (71.2%). The majority of the observed associations were positive (*n* = 86, 56.2% in men and *n* = 82, 53.6% in women). The pattern of node clustering indicates that problematic online gaming and gambling are distinct conditions. The connections between the two conditions were different for men and women. The network comparison test demonstrated significantly higher global expected influence in men compared to women (8.43 vs. 8.20, S = 0.23, *p* = 0.009). However, the results of the network invariance test did not reach statistical significance (M = 0.37, *p* = 0.059). 

Table 2 shows the 10 top-ranked, strongest connections in men’s and women’s networks (for all connections, see Supplementary Tables S1 and S2). All of them appeared within problematic online behaviors. In men, the strongest edge was between nodes GBL4 (“borrowing money/selling anything to gamble”) and GBL8 (“financial problems because of gambling”) (*r* = 0.38), while in women, the strongest one appeared between nodes GBL1 (“betting more than afforded to be lost”) and GBL2 (“tolerance symptoms of gambling”) (r = 0.58). In turn, the connections between nodes representing both conditions had very weak correlation coefficients. The strongest one was between GAM5 (“loss of previous interests due to gaming”) and GBL6 (“health consequences of gambling” in men (r = 0.05) as well as between GAM5 (“loss of previous interests due to gaming”) and GBL7 (“being criticized because of betting or being told about gambling problem”) in women (r = 0.09). 

**Figure 2:**
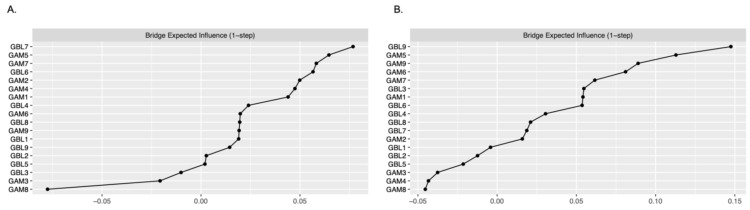
The bridge expected influence metrics in men (**A**) and women (**B**).

The analysis of bridge expected influence (Figure 1A,B and Figure 2) revealed different bridge nodes in men and women. In men, the bridge nodes were GBL7 (“being criticized because of betting or being told about gambling problem”), GAM5 (“loss of previous interests due to gaming”), GAM7 (“deceiving other people because of gaming”), and GBL6 (“health consequences of gambling”). In turn, among women, the bridge nodes were GBL9 (“feeling guilty because of gambling”), GAM5 (“loss of previous interests because of gaming”), GAM9 (“social consequences of gaming”), and GAM6 (“continued gaming problems with other people”). 

### 3.3. The Analysis of DAGs

**Figure 3:**
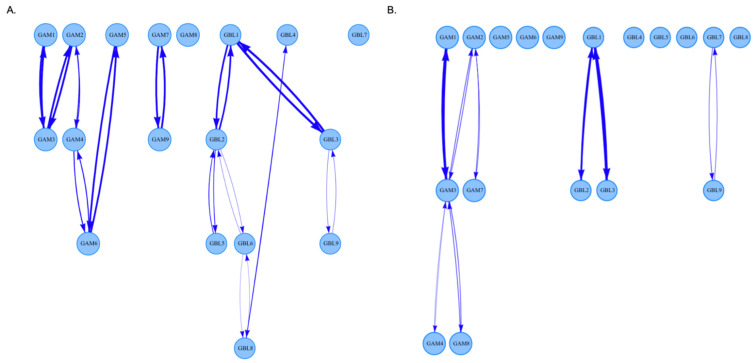
Directed acyclic graphs (DAGs) showing directed associations between the symptoms of problematic online gaming and gambling in men (**A**) and women (**B**). Visualized connections represent those with a threshold > 0.5 (i.e., the associations observed in more than 50% of models).

Directional associations observed in men and women are shown in Figure 3 and Table 3. No connections between problematic online gaming and gambling were observed, with the significance threshold set to >0.5. All connections in both conditions were most likely bidirectional. Importantly, a greater number of influential connections was found in men (*n* = 13 for males vs. *n* = 8 for females). 

## 4. Discussion

The first finding of this study is that both POBs are weakly related to each other, suggesting that they are rather distinct constructs. In addition, in the Bayesian network, there were no arcs connecting the symptoms of both conditions. This means that their unique elements provide evidence to justify their classification as separate diagnostic constructs, despite the general similarity of their diagnostic criteria. This supports the notion that, despite the inclusion of behavioral addictions under the same diagnostic umbrella, they represent different diagnostic entities [18,49,55,64]. Recent studies support the theory that POBs are multiple forms of specific disorders rather than clinical manifestations of a single disorder, i.e., Internet use disorder [68,69,70]. These observations are in agreement with those from other studies using network analysis [18,53,71]. Edge weight coefficients representing both conditions were very weak, making it difficult to determine which symptoms were more likely to account for comorbidity.

Nevertheless, research shows that problematic online gambling and problematic online gaming coexist with each other [21,28,30,72,73]. For example, in the study by Rojas et al., the comorbidity rate was estimated to be 14.5% [8]. However, comorbidity rates need to be interpreted with caution [74]. This is due to the lack of a gold standard procedure to diagnose POBs, and thus, various cut-off scores based on different psychometric instruments [17,75,76]. Previous studies on the comorbidity of gambling and gaming disorders have not provided evidence on the direction of causality within these comorbidity patterns. Zarate et al. showed the greatest centrality for problematic online gambling, which means that it is most likely to lead to the occurrence of other POBs [18]. However, for estimating the directionality of change, Bayesian networks seem to be more useful. In case of our study, we failed to find any arcs linking symptoms of problematic online gaming and gambling. In addition, the results within both POBs suggest that specific symptoms are bidirectionally associated. However, it is important to note that cross-sectional data, although analyzed using Bayesian networks, cannot clearly inform about the direction of causality.

It is noteworthy that most of the research to date, specifically on online gambling, has been focused on men [13]. Therefore, our study might be considered valuable in the context of gender differences. Men and women were found to differ with respect to bridge nodes, with a significantly higher global expected influence on men compared to women. In addition, the number of specific connections was higher in men than in women in the analysis of Bayesian networks, which is in line with previous findings of network analysis studies [55]. These observations indicate that men might be more likely to develop a full spectrum of symptoms representing problematic online gambling and gaming. This is in agreement with the observation that men are generally more susceptible to developing addictions. They are also more likely to show greater impulsivity and its consequences [31]. Moreover, men are more likely to engage in risky behaviors and expect pleasure from them; they are also less likely than women to anticipate the negative consequences of such behaviors [37]. In addition, studies have suggested lower rates of seeking help for gambling problems in men [32]. A number of these characteristics influence men to report higher rates of both substance use and behavioral disorders, and thus, to be at a greater risk of comorbidity. However, it has also been hypothesized that this phenomenon may be related to psychosocial rather than biological factors. Men are more likely to engage in gaming and gambling because of the greater social acceptability of gaming and gambling entertainment among them [17,35]. 

The most strongly interconnected symptoms also differed with respect to gender. In both men and women, the edges of problematic online gambling were stronger than those of problematic online gaming. Concerning the latter, the strongest edge was found between “deceiving other people because of gaming” and “social consequences of gaming”; however, this connection was still very weak. For problematic online gambling, the strongest edge was found between “borrowing money/selling anything to gamble” and “financial problems because of gambling”. The implication is that men may be more likely to lose control of their gambling in the context of financial problems. In fact, this is consistent with previous research reporting that men are more likely to participate regularly and more frequently in the most addictive forms of gambling and to gamble with a higher expenditure [77,78]. This pattern of continuous gambling is associated with an increased risk of financial problems [79]. Moreover, the fact that pathological gambling progresses more rapidly in women means that men need to increase the intensity of their addictive behavior in order to achieve the desired effect, in this case, by playing with larger amounts of money, which escalates financial problems [80]. However, these findings should be interpreted with caution due to the cross-sectional design that limits conclusions about causality.

In women, the strongest edge appeared between “betting more than afforded to be lost” and “tolerance symptoms of gambling”. This indicates that women are more likely to lose control over playing and also develop tolerance more rapidly. The reason for this phenomenon may lie in the motivation behind engagement in these activities with respect to gender. While men are more likely to choose gambling or gaming as a form of entertainment, women are more likely to struggle with emotional distress and other mental disorders [32]. This may make them more vulnerable to losing control. The faster acquisition of tolerance in women may also be related to ‘telescoping’—the phenomenon in which the activity is taken up later in life, and the level of engagement rapidly becomes disrupted [81]. Indeed, women tend to be older at the onset of gambling and might progress more rapidly to problematic gambling [31]. Research data on offline gambling confirm that men are significantly more likely than women to report ever having gambled. However, when considering only gamblers, women were found to be at a greater risk of problematic gambling or progression to gambling disorder [82]. In conclusion, women are observed to move through the various stages of addiction more quickly than men from the initial exposure to addiction. This also suggests that women’s problem behaviors are more similar in structure to substance use disorders, as shown in the study by Lucas et al. [51].

Polish society, the sample of which was used for this study, is considered collectivistic [83]. However, in terms of both online gambling and gaming rates, it is more in line with developed individualistic European countries, which have lower prevalence rates than collectivistic Asian countries [84]. Indeed, in a cross-cultural study by Lopez-Fernandez et al., the rate of online gambling in Poland was the lowest across all 15 countries surveyed. It is also worth noting that Poland is a religious country compared to other European countries, and in such communities, gambling rates are particularly low [85]. However, it is worth noting that the emergence of online subtypes of gambling and gaming has changed the demographic distribution of these disorders [31]. More and more, especially young women, engage in these activities due to the changing profile of psychosocial factors and increased feelings of insecurity among women in relation to Internet use, especially in developed countries with less conservative societies [38]. Nevertheless, women who are addicted to both substances and behaviors still experience higher levels of stigma than men. This observation, together with less social support in their decision to quit, might indicate that women are more isolated and at a greater risk of relapse than men [35].

There are various limitations to the present study that need to be highlighted. First, our sample consisted of young adults with no history of psychiatric treatment. Therefore, its representativeness and potential to generalize the findings might be limited. Although the age groups included in our analysis are those at risk of developing POBs, it does not cover the full spectrum across all age groups, for example, the gambling behavior of older people, children, and adolescents. Although the exclusion of individuals with a psychiatric treatment history may reduce confounding, there is a risk that those who are significantly involved in POBs have significantly increased psychopathological symptoms and receive psychiatric treatment for this reason. Second, the study was based on data from self-reports, which are common in measuring POBs in research. These reports are potentially biased, as clinical validation was not carried out. Third, the study was cross-sectional; thus, the ascertainment of any causal relationships between problematic online gaming and gambling should be approached cautiously. Fourth, the approach of indicating 10% of the nodes with the highest bridge centrality might be perceived as arbitrary. However, specific and robust approaches to define the most central nodes in a network have not yet been developed. Finally, our study did not predefine the option of selecting other gender identities. However, it is likely that these respondents would serve as a minority in the total sample. Therefore, the results of a network analysis of the symptoms in this subgroup would likely show low stability.

## 5. Conclusions

To summarize, the present study indicates that problematic online gambling and problematic online gaming are distinct diagnostic constructs. The dynamics of specific symptoms might be different according to gender. Our observations suggest that men might be more likely to develop a full spectrum of symptoms representing both problematic online gambling and gaming. Moreover, men might be more prone to develop financial problems, while women might be more likely to lose control and develop tolerance. Altogether, these findings indicate that the development of problematic online gaming and gambling may show different trajectories in men and women. These trajectories and symptom dynamics need to be taken into consideration while proposing specific therapeutic interventions. The findings also deliver some perspectives for future studies of POBs that need to consider the use of network analysis approaches to longitudinal data from various clinical and non-clinical populations in order to better inform about causality and clinical implications. 

## Figures and Tables

**Table 1:** General characteristics of the sample (*n* = 1441).

	Mean ± SD or *n* (%)
Age, Years	29.5 ± 6.3
Gender	
Men	701 (48.6)
Women	740 (51.4)
Education	
Primary	91 (6.3)
Vocational	109 (7.6)
Secondary	617 (42.8)
Higher	624 (43.3)
Employment	
Unemployed	168 (11.7)
Part-time work	193 (13.4)
Full-time work	763 (52.9)
Student	312 (21.6)
Other	5 (0.3)
Place of residence	
Rural	523 (36.3)
Urban (up to 50,000 inhabitants)	331 (23.0)
Urban (50,000–150,000 inhabitants)	161 (11.2)
Urban (150,000–500,000 inhabitants)	178 (12.4)
Urban (>500,000 inhabitants)	248 (17.2)
Income	
<750 USD	331 (23.0)
750–1250 USD	565 (39.2)
1250–1750 USD	211 (14.6)
1750–2500 USD	69 (4.8)
>2500 USD	33 (2.3)
Declined to answer	232 (16.1)
Marital status	
Married	475 (33.0)
Informal relationship	349 (24.2)
Single	275 (41.1)
Divorced	25 (1.7)
IGDS9-SF, score	16.5 ± 6.4
IGDS9-SF, positive screening	30 (2.1)
PGSI, score	5.5 ± 5.4
PGSI, positive screening	86 (6.0)

Note: IGDS9-SF, The Internet Gaming Disorder Scale–Short-Form; PGSI, the Problem Gambling Severity Index.

**Table 2:** Top 10 partial correlation coefficients in the network of problematic online gaming and gambling among males and females.

Gender	Edge	r
Men	GBL4–GBL8	0.38
	GBL6–GBL8	0.32
	GBL1–GBL2	0.31
	GBL1–GBL3	0.31
	GBL2–GBL5	0.30
	GAM7–GAM9	0.29
	GAM2–GAM3	0.28
	GBL8–GBL9	0.26
	GAM1–GAM8	0.26
	GAM1–GAM3	0.23
Women	GBL1–GBL2	0.58
	GBL5–GBL6	0.40
	GBL7–GBL9	0.37
	GBL6–GBL8	0.28
	GAM7–GAM9	0.27
	GBL7–GBL8	0.26
	GBL1–GBL3	0.26
	GAM2–GAM3	0.25
	GAM3–GAM8	0.22
	GBL6–GBL7	0.20

**Table 3:** Strength of connections between symptoms of problematic gaming and gambling in males and females.

Similarity between Men and Women	Effect	Strength	
		Males	Females
Similar in men and women	GAM1 → GAM3	0.90	0.97
	GAM2 → GAM3	0.91	0.75
	GBL1 → GBL2	0.86	0.89
	GBL1 → GBL3	0.99	0.96
	GAM3 → GAM1	0.90	0.97
	GAM3 → GAM2	0.91	0.75
	GBL2 → GBL1	0.86	0.89
	GBL3 → GBL1	0.99	0.96
Identified only in men	GAM2 → GAM4	0.70	–
	GAM4 → GAM6	0.72	–
	GAM5 → GAM6	0.89	–
	GAM7 → GAM9	0.90	–
	GBL2 → GBL5	0.66	–
	GBL2 → GBL6	0.57	–
	GBL6 → GBL8	0.51	–
	GBL4 → GBL8	0.62	–
	GBL3 → GBL9	0.57	–
	GAM4 → GAM2	0.70	–
	GAM6 → GAM4	0.72	–
	GAM6 → GAM5	0.89	–
	GAM9 → GAM7	0.90	–
	GBL5 → GBL2	0.66	–
	GBL6 → GBL2	0.57	–
	GBL8 → GBL6	0.51	–
	GBL8 → GBL4	0.62	–
	GBL9 → GBL3	0.57	–
Identified only in women	GAM2 → GAM7	–	0.73
	GAM3 → GAM4	–	0.68
	GAM3 → GAM8	–	0.74
	GBL7 → GBL9	–	0.74
	GAM7 → GAM2	–	0.73
	GAM4 → GAM3	–	0.68
	GAM8 → GAM3	–	0.74
	GBL9 → GBL7	–	0.74

## Data Availability

The raw data supporting the conclusions of this article will be made available by the authors upon request.

## Supplementary Materials

The following supporting information can be downloaded at: https://www.mdpi.com/article/10.3390/brainsci14090929/s1, Table S1. Edge weights in men. Table S2. Edge weights in women.
