# Supplementary material for: Understanding the Emergence of Comorbidity between Problematic Online Gaming and Gambling: A Network Analysis Approach
Source: Brain Sci. 2024 Sep 18;14(9):929. doi: 10.3390/brainsci14090929 (PMC11444146; doi:10.3390/brainsci14090929)
Supplement: Supplementary file 1 [file brainsci-14-00929-s001.zip › brainsci-3186286-supplementary.pdf]

Supplementary Materials

Table S1. Edge weights in men.

	GAM1	GAM2	GAM3	GAM4	GAM5	GAM6	GAM7	GAM8	GAM9	GBL1	GBL2	GBL3	GBL4	GBL5	GBL6	GBL7	GBL8
GAM2	0.102																
GAM3	0.231	0.275															
GAM4	0.090	0.218	0.183														
GAM5	0.067	0.031	0.062	0.145													
GAM6	0.089	0.087	0.039	0.156	0.232												
GAM7	0.008	0.105	0.110	0.065	0.110	0.174											
GAM8	0.259	0.113	0.116	0.065	0.000	0.026	0.060										
GAM9	0.094	0.066	0.055	0.106	0.169	0.078	0.291	0.000									
GBL1	0.000	0.000	0.035	0.000	-0.010	0.015	-0.026	0.000	0.000								
GBL2	0.012	0.000	0.000	0.000	0.000	0.000	0.000	-0.017	0.000	0.312							
GBL3	0.000	0.000	0.000	0.000	0.000	0.000	0.000	0.026	-0.026	0.312	0.143						
GBL4	0.009	-0.013	-0.029	0.000	0.070	0.011	0.000	-0.021	0.000	0.006	0.000	-0.013					
GBL5	0.000	0.028	0.040	0.000	-0.042	0.000	0.036	0.000	-0.022	0.059	0.295	0.078	0.227				
GBL6	0.000	0.000	-0.007	0.000	0.048	-0.017	0.037	-0.044	0.042	0.070	0.165	-0.058	0.050	0.020			
GBL7	-0.010	0.038	-0.030	0.054	-0.003	0.000	0.007	0.000	0.022	0.117	0.075	0.064	0.063	0.111	0.210		
GBL8	0.016	0.000	-0.041	0.000	0.036	0.015	0.007	0.000	0.000	0.033	0.055	-0.029	0.381	0.000	0.322	0.026	
GBL9	0.025	0.000	0.000	0.000	0.015	0.000	0.000	-0.028	0.000	0.114	-0.055	0.277	0.000	0.279	0.179	0.048	0.263

Table S2. Edge weights in women.

	GAM1	GAM2	GAM3	GAM4	GAM5	GAM6	GAM7	GAM8	GAM9	GBL1	GBL2	GBL3	GBL4	GBL5	GBL6	GBL7	GBL8
GAM2	0.156																
GAM3	0.192	0.249															
GAM4	0.186	0.159	0.163														
GAM5	0.098	0.052	0.138	0.135													
GAM6	0.065	0.068	0.076	0.164	0.133												
GAM7	0.073	0.204	0.000	0.050	0.004	0.139											
GAM8	0.146	0.000	0.221	0.094	0.026	0.157	0.045										
GAM9	-0.024	0.082	0.052	0.000	0.195	0.089	0.271	0.060									
GBL1	0.018	0.000	0.027	-0.010	0.000	0.008	0.000	-0.047	0.000								
GBL2	0.000	-0.031	0.018	0.029	0.014	0.000	-0.019	-0.013	-0.009	0.582							
GBL3	0.000	0.000	0.000	0.000	-0.036	0.034	0.019	0.048	-0.010	0.262	0.186						
GBL4	0.020	-0.035	0.000	0.010	0.013	0.000	0.068	-0.046	0.000	0.144	-0.042	0.009					
GBL5	0.010	0.000	0.000	0.000	0.000	0.030	-0.033	0.000	-0.029	0.125	-0.077	0.174	0.067				
GBL6	0.006	0.000	0.000	-0.033	0.000	0.000	-0.028	-0.008	0.117	0.096	-0.143	-0.124	0.122	0.402			
GBL7	0.000	-0.052	0.000	0.000	0.087	0.009	-0.024	0.000	0.000	0.000	-0.081	0.000	0.085	0.000	0.204		
GBL8	0.000	0.005	0.000	0.000	0.000	0.000	0.000	0.000	0.016	0.000	0.354	-0.020	0.000	0.198	0.278	0.264	
GBL9	0.000	0.128	-0.083	-0.039	0.036	0.000	0.079	0.021	0.004	0.076	-0.018	0.101	0.158	0.136	0.000	0.369	0.140
